# Conducting research with young people at the margins – lessons learnt and shared through case studies in Cambodia, India, Sweden and Zambia

**DOI:** 10.1186/s12889-022-14427-8

**Published:** 2022-11-25

**Authors:** Frida Jonsson, Puthy Pat, Chama Mulubwa, Bhoomikumar Jegannathan, Kaaren Mathias

**Affiliations:** 1 Department of Epidemiology and Global Health, Umeå University, Umeå, SE Sweden; 2 Arctic Research Centre (Arcum) at Umeå University, Umeå, Sweden; 3 Center for Child and Adolescent Mental Health (Caritas-CCAMH), Krŏng Ta Khmau, Cambodia; 4 School of Public Health, University of Zambia, P.O. Box 50110, Lusaka, Zambia; 5 Burans, Herbertpur Christian Hospital, Uttarakhand, India; 6 Te Kura Mātai Hauora School of Health Sciences, University of Canterbury, Christchurch, New Zealand

**Keywords:** Young people, Marginalisation, Participatory research, Global health, Equity

## Abstract

Building on the value of engaging with and enabling the participation of marginalised young people in research, the aim of this article was to profile practical and procedural issues faced when conducting studies with young people who experience some form of marginalisation. Drawing on observations and research experiences from four diverse case studies involving young people who were either imprisoned in Cambodia, living in informal urban communities in North India, residing in rural northern Sweden or attending school in rural Zambia, learnings were identified under three thematic areas. Firstly, a need exists to develop trusting relationships with stakeholders, and especially the participating young people, through multiple interactions. Secondly, the value of research methods that are creative and context sensitive are required to make the process equitable and meaningful for young people. Thirdly, it is important to flatten power relations between adults and young people, researchers and the researched, to maximise participation. These findings can inform future youth research in the field of global public health by detailing opportunities and challenges of engaging in research with young people on the margins to promote their participation.

**Supplementary Information:**

The online version contains supplementary material available at 10.1186/s12889-022-14427-8.

## Introduction

Promoting the health and well-being of young people is a growing priority in the fields of health research, policy and practice [1]. This focus recognises that while young people typically live fairly healthy lives compared to adults and elderly populations, problems such as mental ill health, unintentional injuries, interpersonal violence and substance misuse are common within this demographic segment [2]. The growing international awareness about the health risks for young people builds on the fact that adversity during youth and young adulthood can have long-term implications [3] and that those disadvantaged by socioeconomic background, gender or residential location have poorer health than their privileged peers [4]. In addition, the COVID-19-pandemic and subsequent breakdowns in systems of health and social care have clearly shown the fragility of young people’s well-being, especially among those who were already marginalised [5]. Engaging with and enabling the participation of young people on the margins of society is thus key to ensure that services and supports are accessible, relevant and feasible.

As a means to promote health equity, contribute to social change and ultimately improve the situation of young people who experience marginalisation, participatory research methodologies have been identified as key to increasing equitable outcomes [6–16]. As synthesised by Anyon et al. [12], the aspiration to conduct research ‘with’ as compared to ‘on’ young people, integrates both core principles and processes. The principles state that participatory youth research should be grounded in the lives and lived experiences of young people, be driven by a focus on co-construction with meaning created in collaborative youth/adult partnerships and be emancipatory, respectful and aimed at transforming the situation of young people and their communities for the better [13]. Participatory youth research should also be conducted through processes that ensure more equal power relations so that young people can govern and gain influence over projects. It should also integrate research with action to allow young people to practise their strategic thinking, apply their skills and build alliances with stakeholders [14].

At the general level, participatory youth research aligns with a human rights approach and Article 12 of the Convention on the Rights of the Child, which states that young people should be able to have a say in and control over aspects relevant to their lives [17]. However, while there is a strong case for considering marginalised young people as resourceful agents rather than as passive recipients, involving them in the research process is not always easy. Young people can at times ‘exploit, appropriate, redirect, contest or refuse participatory techniques’ ([18] p. 137) and when done poorly, the researcher(s) may further regulate rather than empower young people by requiring certain types of participation. In this regard, Fox [8] has discussed the challenges of unequal power relations and the limiting discourses of academic research. She suggests moving beyond ‘researcher’ and ‘researched’ subject positions to achieve meaningful youth participation that changes the status quo by creating spaces for resistance and social transformation.

Against this backdrop, the current ‘research in practice’ article was developed to profile practical and procedural issues faced when conducting research in four diverse settings globally with young people who experience some form of marginalisation. It emerged from dialogs between us, the authors, as part of our engagements as PhD students and researchers at the Department of Epidemiology and Global Health, Umeå University (Sweden), where we found a strong shared interest in youth research. Based on this shared interest, we developed a proposal for a joint workshop session at the Sixth Global Symposium on Health Systems Research in 2020 with a focus methods that could improve the research quality when engaging with marginalised young people. During the process of preparing for and presenting during the workshop, we identified common opportunities, challenges and critical reflections. Since future youth research in the field of global public health could potentially benefit from these insights, we resolved to try to work on these together in the form of an article.

Based on an emergent design, the analysis through which we developed the lessons presented in this article comprised the following steps. We started with an iterative process involving several virtual meeting to discuss and define the aim of the work. The first author (FJ) offered to lead the process of profiling practical and procedural issues faced when conducting research with marginalised young people. We then collectively developed a template (Supplementary Table 1) to summarise and compile information about our projects. The process continued to develop key lessons, involving both challenges and opportunities, through the identification of themes in the material as a whole as well as a cross case analysis together led by FJ and KM. Through the dynamics of writing up the ‘lessons learnt’ and continued discussions in the team, refinements were made to the template to draw more information about power relations in the different projects since this emerged as an important theme.

Before presenting the three thematic areas that were developed from the analysis, we give a brief overview of the research projects (‘cases’) from which we draw our experiences.

## Case descriptions

To contextualise the lessons learnt and shared in this article, below we present some details about our research conducted with young people who were imprisoned in Cambodia, living in informal urban communities in North India, residing in rural areas of northern Sweden and attending school in rural Zambia. Rather than focusing on the findings, the information presented below outline the aims, participants and methodologies of the research. Table 1 summarises the four projects and in all cases, researchers were nationals or long-term residents in the particular country and spoke the local language.

**Table 1:** Overview of the four research projects (‘cases’)

	**Cambodia**	**India**	**Sweden**	**Zambia**
Host organisation	Center for Child and Adolescent Mental Health (Caritas-CCAMH)	Burans, a mental health initiative of Herbertpur Christian Hospital	Umeå University	The University of Zambia, School of Public Health
Setting	Four prisons representative of the regions in Cambodia	Three informal urban communities in North India	Five rural communities in northern Sweden	Four rural schools in the Central Province of Zambia
Time	January 2018–August 2019	March 2019 to March 2020	August 2019–January 2020	August–November 2018
Study aim	To understand the mental health profiles and needs among young prisoners and to ‘give voice’ to them in shaping the prison mental health system	To explore gender relations for young people living in urban low-income communities	To explore collective imaginaries of caring landscapes for rural youth	To analyse constructions of young people’s sexualities and sexual health and the consequences of these discourses for their sexual reproductive health and rights
Participants	Young people aged 15–24 years involved in the cross-sectional survey (young men) and in the six FGDs (young men and women)	Young men and women aged 12–19 years of age	Young people aged 15–27 years of age with diverse ethnicity, gender, functionality and sexuality	Male and female students aged 16 and older who had previously participated in an intervention aimed at increasing comprehensive sexual knowledge
Methodology	Mixed methods research	Qualitative study nested within a randomised controlled trial	Concept mapping (mixed methods research)	Qualitative study
Methods	Quantitative cross-sectional survey and qualitative focus group discussions	Multiple qualitative data modelled on participatory rural appraisal collected by both youth peers and researchers	Qualitative interviews and focus group discussions combined with a workshop and multivariate statistical methods	Photo elicitation in combination with in-depth interviews and focus group discussions

### Giving voice to young prisoners in Cambodia: a mixed methods study on mental health

The aim of this mixed methods study was to give voice to imprisoned young people and examine their mental health challenges and access to psychosocial care within the prison setting. Young men who were incarcerated (convicted and under appeal) were invited to participate in surveys while young prisoners of both sexes were invited to participate in focus group discussions (FGDs). All participants were recruited by the researchers through the prison authorities and gave informed consent. The survey instruments included Youth Self-report [19] and Attitude Toward Suicide questionnaires [20], which were field-tested. The collection of survey data was challenging because most of the young people were pre-literate and had limited comprehension of questionnaires related to mental health status. The questionnaires were therefore administered by the researchers, and to improve understanding, visual illustrations were used to facilitate responses. The FGDs were conducted with young people through open circles with careful seating arrangements away from prison personnel, who were asked to keep their distance in order to maximise confidence and trust in the limited privacy setting of a prison. The study emerged from the need to ‘listen to young prisoners’ on issues related to their overall health, particularly mental health [21, 22]. The results will thus be shared through national-level consultative workshops with planners and policymakers, enabling the voice of the young prisoners to be heard to promote reform and development of prison mental health services. The survey data were analysed using multivariable regression analysis and have been published in a peer-reviewed journal [23], while the FGD data were analysed using thematic analysis [24]. The write-up of this study is underway.

### Examining gender relations among young people in informal urban communities using participatory data collection: a qualitative study in North India

The aim of this study was to examine how young people as data collectors could contribute deeper contextual understandings of gender relations and youth resilience. This qualitative study was nested within a larger randomised controlled trial evaluating Nae Disha (New Pathways), a youth resilience and mental health intervention [25]. The participating young people were all residents of low-income slum communities and the majority described that they had lived experienced of social exclusion, for example, because of mental health problems, substance or alcohol abuse (particularly among young men) and unemployed parents or living in low income households. In weekly group meetings, young people followed structured modules addressing positive youth development and equal relations. The data collection processes was modelled on participatory rural appraisal approaches [26] to include the following: i) several workshops facilitated by young people under the age of 30 using methods such as role plays, drawing, storytelling and group discussions to gather data; ii) FGDs facilitated by youth peers; iii) in-depth interviews and FGDs facilitated by a researcher; and iv) ethnographic observations. The pictures, written outputs from young people and transcripts from interviews and FGDs (translated verbatim from Hindi to English) were analysed using thematic analysis [24]. A paper analysing these data is currently being written up for wider dissemination.

### Collective imaginaries of caring landscapes for rural youth: a concept mapping study in northern Sweden

The aim of this concept mapping study was to explore collective imaginaries of caring landscapes for rural youth, focusing on what such landscapes ideally should look like and how various strategies could help to realise the visions. Participating in the study were young people marginalised by their rural area of living as well as professionals and policy makers living and working in northern Sweden. Compared to a more ‘traditional’ concept mapping procedure where participants are often merely supported by the researchers in visualising and reporting their ideas [27], the project was characterised by an active co-construction of knowledge, with insights being generated in dialogue between participants and researchers based on three phases of data collection and analysis. These phases included (i) a secondary analysis of data collected for another study conducted by the team [28], (ii) a one-day workshop where young people and professionals were invited to brainstorm, and (iii) a sorting activity where the workshop participants were asked to group statements identified in the brainstorming in ways that appeared meaningful for them using an online software. The focus of this project was to prioritise and highlight the young people’s experiences, and during the first phase, a large number of young people (63 individuals) from diverse backgrounds were engaged in the research. However, in the workshop and sorting activity, only a few young people participated (6 and 3, respectively), which was a substantial limitation of the research. The qualitative data from phases one and two were analysed using thematic analysis [24], while the sorted data from phase three were analysed using multivariate statistical methods and identification of thematic clusters. The findings have been published in a peer-reviewed journal [29] and communicated to participants and the public through short lay language summaries.

### Using photo elicitation to explore discourses on sexuality and sexual health among young people in rural Zambia

The aim of this photo elicitation interview (PEI) study was to gain insight into discourses on sexuality and sexual health, as well as the consequences of these discourses for young people’s exercise of their sexual and reproductive rights. The combination of PEI with individual and group interviews to discuss sexuality with young people has been advocated for in previous research as a means to triangulate responses on young people’s sexuality [30]. In this approach, the four stages of PEI (preparation, taking photographs, interviews and post-interviews) described by Overmars-Marx et al. [31] were tailored to suit the study. Although the study may not be considered fully participatory, many steps were taken to engage young people and alleviate power imbalances. Prior to commencing the PEI, young people were informed about the study objectives and use of a camera, including ethics related to taking photos. During the photo-taking stage, participants equipped with cameras were invited to take pictures of any part of their lives that could illustrate how they constructed, understood and viewed sexuality and sexual health. In the interview and post-interview stage, the researchers used the pictures to start the discussions around sexuality. Further information on the PEIs were elicited during individual and group interviews using an open-ended interview guide with thematic areas previously discussed with the participants during the workshop. Both the qualitative (textual) and visual data were analysed using the analytical approach of interpretative repertoires, which is rooted in discursive psychology [32]. After analysing constructions of young people’s sexualities and sexual health and the consequences of these discourses for young people exercising their sexual and reproductive rights, the results were published in a peer-reviewed journal [33].

## Lessons learnt and shared

While the four case studies presented used different methodological approaches to address diverse research aims among marginalised young people, we identified several common opportunities and challenges. These are outlined below to share observations and lessons to inform future studies.

### Meet often to build trusting relationships

The combination of various methods (see Table 1) coupled with multiple personal contacts and repeated meetings with the participating young people and practitioners working with (or for) them, allowed researchers to raise awareness about the project and to build trusting relationships.

Frequent and ongoing interactions with marginalised young people were key to gaining their trust and engaging them in the research. In Cambodia, the prison system in itself creates mistrust of the authorities, and the researchers took extra efforts to overcome these barriers, such as taking senior prisoners into their confidence to facilitate the data collection process, a peer facilitated model [34]. In India, repeated group meetings and workshops with the researchers and the young people allowed peer friendships to develop between participants and with the research team. With growing relationships, the participating young people started to engage in dialogue, which in combination with meeting in smaller groups (e.g. of three to five people), seemed to contribute to more open and authentic discussions. Similar experiences emerged in the Zambian context, where multiple interactions during the photo-taking stage led to young people feeling comfortable about discussing experiences and perceptions related to sex and contraceptive use. This sense of safety was also aided by the exclusion of teachers and other school staff from the meetings, an emphasis on confidentiality of information and by respecting individual opinions shared or discussed.

We identified that particularly for young people who were highly marginalised and hard to reach, it was important to build relationships with stakeholders who could be identified as gatekeepers. Experiences from the Cambodian, Zambian and Swedish case studies illustrated how, similar to Russell [35], a long-term approach might not only require frequent and ongoing interactions with the young people but necessitate repeatedly consulting various (adult) stakeholders before youth participants could even be identified or approached. In Cambodia, where the participating young people were severely disempowered due to their incarceration, the process of giving them voice and the opportunity to engage in the research meant that the prison directorate first had to be convinced of the value of the study. Building on existing collaborations and previous research [36], through multiple meetings with the administrators, who were initially sceptical and therefore reluctant to initiate research in the prison setting, the team eventually gained the approval and contacts necessary to approach the young people in prison. In the Zambian case study, similar experiences came across, where the research team was only allowed to proceed with PEI after the school authorities were convinced that the research would not teach ‘inappropriate’ lessons on sexuality to the young people. Young people were also hard to reach in the Swedish case study without the help of adult gatekeepers. Here, strategies such as several site visits and repeated personal contacts with stakeholders allowed the team not only to familiarise itself with the setting but to identify professionals who, in turn, could provide access to young people.

In all four cases, it was important not to rush or force the building of relationships. Instead, time and resources (both funding and skills) were needed, particularly to gain the trust of young people. This need may have been necessary because all four cases included young people who had experienced structural disadvantages linked to their place of residence, incarceration or socio-economic status, which may make their barriers to participation especially high [37]. In addition, young people on the margins of society are often distrustful of authorities and reluctant to engage with institutions due to various oppressive structures, meaning that a focus on developing trusting relationships is key to promoting their participation in research [11].

### Use creative and context-sensitive research methods

The four case studies pointed to the value of research that used creative and context-sensitive approaches. Specifically, they illustrate how practical, playful and peer-based methods that move beyond 'traditional' ones, such as semi-structured interviews, can be a pragmatic and ethical way of conducting research, especially in vulnerable communities [7, 9, 38].

In India, young people co-facilitated FGDs and worked as data collectors among peers in interactive workshops. Participants shared their experiences by telling stories or drawing pictures. Data collection in Zambia, in turn, involved young people being invited to take, select and discuss photographs to depict their perceptions, experiences and unique discourses on sexuality. In line with youth study scholars who have argued for the need to adopt task-based approaches that are youth-led, fun and informal [9, 15], the use of such methods appeared to enhance the young people’s enthusiasm for and engagement in the research. Innovative techniques comprising visual illustrations were also used to depict questionnaire responses from young prisoners in Cambodia, who were not only disempowered because of their incarceration but also had low literacy. While this context-sensitive approach addressed the needs of young people limited by their ability to comprehend and respond to written questions, the use of a software in the third phase of the research in Sweden was insufficient to engage rural young people in the research. This means that while public discourses often depict young people as ‘digital natives’ [39], the mere adaptation to online solutions was not sufficient to promote their participation.

When situated within a participatory framework, practical, playful and peer-based approaches can stimulate dialogue and a frank sharing of opinions that are not censured or interpreted by adults while also having the potential to reduce unequal power differentials [16, 40, 41]. In this regard, the peer-to-peer interviews conducted in India seemed to expand the participating young women’s worldview of more equal gender relations, leaving them feeing excited and enthused to negotiate for greater freedom of movement in their families. The use of photo elicitation in Zambia also allowed the researchers to move from discussing the pictures, which typically represented something familiar and fun, to more serious and sensitive questions and probes into sexuality. The benefits of using pictures in this way align with research that shows how the use of photos can help to reduce awkwardness experienced during traditional interviews [42, 43]. When conducting face-to-face fieldwork in Sweden, the researchers also disclosed aspects of themselves, using more ‘informal language’ in dialogue with the young people. Similar to Conolly [9], this approach was adopted to set the discussions off on a more equal footing and to allow for in-depth and sometimes sensitive questions to be asked about their lives.

While our case studies indicate that creative and context-sensitive methodologies can positively impact on the young people’s research experience while improving the outcomes of the research by making the process more equitable and purposeful, such approaches do not offer a solution per se to their marginalisation. In fact, transitions towards a more inclusive and equitable society will entail moving beyond a downstream focus on research participation in to an ‘upstream’ policy focus on reducing social, economic and health-related inequalities, where collective responses and actions for justice that seek broader societal change can contribute to youth development more generally [13].

### Flatten typical power hierarchies

In addition to the opportunities and limitations of engaging in research with marginalised young people as presented above, this last section presents a number of challenges that constrained our attempts to create knowledge *with* young people *for* young people through collaborative forms of youth participation [11, 12].

Although co-learning whereby young people are involved already in the design of the project and then consistently throughout the research is at the heart of participatory youth research [7], in all our cases the decision-making was not shared between the (adult) researchers and (young) participants. Instead, following the standard routes of research and constraints imposed by traditional academic systems and structures [8, 11], the study protocols were developed by the researchers alone or in collaboration with stakeholders. This meant that adults and academics framed the research questions, chose the methods and controlled the analysis. In Sweden, young people’s hesitancy to participate in the face-to-face workshop and online questionnaire might reflect a resistance to the adultism [11] of adult- and academia-centric approaches [29]. In India, where the research was much more collaborative and ‘youth-centred’, it is more likely that the problems related to engaging young people in later stages of the research stemmed from difficulties in sustaining trusting relations to foster authentic youth–adult partnerships. The regimental structures and ambience of the Cambodian prison system, in which the young people were severely disempowered, limited the opportunities for collaborative participation and also acted as barriers to the participants fully expressing themselves [44]. Considering the value and possibilities of approaching young people directly when the research is conducted in a non-institutional context (see e.g. [45]), the Swedish case study could also have benefitted from moving beyond a standardised recruitment process involving gatekeeping to a more informal one drawing upon local youth networks to more effectively build rapport, develop trust and flatten power relations.

Against this backdrop, pointing to the constraints that shaped our attempts to conduct participatory youth research, we agree with Rodríguez and Brown that the challenge for marginalised young people in shaping and influencing the process ‘hinges not on a lack of voice but a lack of power’ ([13], p. 32). While the approaches employed in the case studies may have moved us, at least partly, away from problematic notions of ‘helping’ young people, our sincere attempts to give voice to marginalised young people have most likely not resulted in immediate emancipation and empowerment [10]. However, following the discussions of both Fox [8] and Teixeira et al. [11], a first step towards developing youth–adult partnerships that have the potential to challenge oppressive systems in academia and society at large is critical self-reflection. As scholars interested in doing research *with* young people that is transformatory and truly grounded in their lives and lived experiences [12], we recognise the concurrent and future need to address the adultism shaping our work through continuous discussions of *how* young people’s involvement may be constrained by structures or simply reflect our assumptions about what is appropriate and possible [8, 11].

## Conclusions

This research-in-practice article describes four case studies seeking to enable the participation of marginalised young people in diverse settings. Through collectively and critically reviewing our own work, we have distilled and presented three key ‘lessons learned’ to inform future youth research in the field of global public health. Taken together, the observations and experiences profiled in this article show how young people’s participation in research constitutes a methodological *principle* to be aimed for rather than being inherent in or bound to specific methods. Most importantly, conducting participatory youth research will entail critical self-reflection and the active positioning of the (adult) researcher(s), who not only will have to be responsive to the needs, wishes and initiatives of young people but will have to be open to new ways of working, to delegating power and to minimising control [11, 16].

## Supplementary Information

**Additional file 1: **Bespoke template to compile details about the projects that provided a basis for the analyses.

## Data Availability

The data collected and analysed for the different projects is not publicly available because it contains sensitive information, but is available from the authors on reasonable request.

## Abbreviations

FDG: Focus group discussions
PEI: Photo elicitation interview
