# Supplementary material for: Conducting research with young people at the margins – lessons learnt and shared through case studies in Cambodia, India, Sweden and Zambia
Source: BMC Public Health. 2022 Nov 25;22:2185. doi: 10.1186/s12889-022-14427-8 (PMC9700999; doi:10.1186/s12889-022-14427-8)
Supplement: Supplementary file 1 — Additional file 1: Bespoke template to compile details about the projects that provided a basis for the analyses. [file 12889_2022_14427_MOESM1_ESM.docx]

# Supplementary material

**Supplementary table 1. Bespoke template to compile details about the projects that provided a basis for the analyses.**

|  | **Cambodia** | **India** | **Sweden** | **Zambia** |
| --- | --- | --- | --- | --- |
| **Background** |  |  |  |  |
| - Host organisation |  |  |  |  |
| - Aim of research |  |  |  |  |
| - Setting |  |  |  |  |
| - Participants |  |  |  |  |
| - Methodology |  |  |  |  |
| - Study design/process |  |  |  |  |
| **About the data** |  |  |  |  |
| - Collection |  |  |  |  |
| - Analysis |  |  |  |  |
| **Dissemination** |  |  |  |  |
| - To academia |  |  |  |  |
| - To policy and practice |  |  |  |  |
| **Key challenges** |  |  |  |  |
| - With research overall |  |  |  |  |
| - Data collection and analysis |  |  |  |  |
| **Key opportunities** |  |  |  |  |
| - With research overall |  |  |  |  |
| - Data collection and analysis |  |  |  |  |
| **Lessons learned** |  |  |  |  |
| - Worked well |  |  |  |  |
| - Could have been done differently |  |  |  |  |
